# Fluorescently Tagged *Verticillium dahliae* to Understand the Infection Process on Cotton (*Gossypium hirsutum*) and Weed Plant Species

**DOI:** 10.3390/pathogens13060442

**Published:** 2024-05-23

**Authors:** Andrew Chen, Sabrina Morrison, Aphrika Gregson, Duy P. Le, Andrew S. Urquhart, Linda J. Smith, Elizabeth A. B. Aitken, Donald M. Gardiner

**Affiliations:** 1 School of Agriculture and Food Sustainability, The University of Queensland, St. Lucia, QLD 4072, Australia; sabrina.morrison@uq.edu.au (S.M.); aphrika.gregson@dpi.nsw.gov.au (A.G.); 2 Queensland Alliance for Agriculture and Food Innovation, The University of Queensland, St. Lucia, QLD 4072, Australia; 3 New South Wales Department of Primary Industries, Orange, NSW 2800, Australia; 4 New South Wales Department of Primary Industries, Narrabri, NSW 2390, Australia; duy.le@dpi.nsw.gov.au; 5 Applied Biosciences, Macquarie University, Macquarie Park, NSW 2109, Australia; 6 EcoSciences Precinct, Department of Agriculture and Fisheries, Dutton Park, QLD 4102, Australia; linda.smith@daf.qld.gov.au

**Keywords:** alternative weed plant host, defoliating and non-defoliating pathotypes, green fluorescent protein, mCherry red fluorescent protein, *Nicotina benthamiana*, plant host and pathogen interactions, reporter genes, vegetative compatibility groups 1A and 2A

## Abstract

Verticillium wilt is a soil-borne disease caused by distinct vegetative compatibility groups (VCG) of the fungus *Verticillium dahliae*. Defoliating (VCG 1A) and non-defoliating (VCG 2A) pathotypes of *V. dahliae* have contributed to yield losses of cotton production in Australia. To study the virulence and the infection process of *V. dahliae* on cotton, two isolates, one representing each VCG, have been transformed with fluorescent protein genes. The transformants maintained their ability to infect the host, and both strains were observed to move through the plant vasculature to induce wilt symptoms. Furthermore, virulence testing suggests that the cotton *V. dahliae* strains can endophytically colonise common weed plant species found in the Australian landscape, and that is contrasted by their ability to infect and colonise native tobacco plants. The fluorescently labelled strains of *V. dahliae* not only allowed us to gain a thorough understanding of the infection process but also provided a method to rapidly identify recovered isolates from host colonisation studies.

## 1. Introduction

Cotton (*Gossypium* L.) is derived from dicotyledonous, perennial shrubs cultivated for their soft fibres that develop around the seeds of mature fruit of the plant [1]. The genus *Gossypium* contains over 50 species, of which 4, which are cultivated globally as annual crops and known as upland cotton, represent 90% of global cotton production [2]. In Australia, upland cotton production comprises an expanding multi-billion-dollar industry that employs over 12,000 people nationwide [3].

*Verticillium dahliae* Kleb. is an asexual, soil-borne fungus affecting over 400 plant species [4]. *Verticillium dahliae* is the primary causal agent of Verticillium wilt in many economically important crop species, including upland cotton [5]. Verticillium wilt is a systemic disease that arises from the colonisation and subsequent occlusion of the host vasculature by the pathogen [6]. Plant symptoms include wilting, stunting, the dropping of foliage, discoloured vascular tissue, and necrotic lesions, leading to plant death [7]. In Australia, Verticillium wilt is reported to cause a reduction in cotton yields in the range of 10% to 62% under pathogen-conducive conditions [8]. The eradication of *V. dahliae* is challenging once it becomes established in the field due to the presence of microsclerotia, a form of resting spore that can remain dormant in soils for years surviving on decaying tissues [9].

Pathogenic strains of *V. dahliae* are generally categorised based on pathotype [10] or race [11]. In cotton, *V. dahliae* pathotypes are described as either defoliating or non-defoliating, depending on the symptoms induced in the host. The defoliating pathotype is generally considered more severe, whilst non-defoliating isolates may vary in disease aggression [12]. Populations within *V. dahliae* can be further classified on a sub-species level based on vegetative compatibility grouping (VCG) [13].

Prior to the 2013/14 cropping season, the only *V. dahliae* pathotype reported in Australian cotton fields was the mildly virulent VCG 4B; however, this changed following the confirmation of the non-defoliating VCG 2A pathotype in New South Wales (NSW) from isolates collected in the 2009/10 season [14]. Chapman et al. (2016) used VCG classification with *nit* mutant testing and molecular assays performed on a set of eight historical isolates dating back as far as the 2009/2010 growing seasons in NSW Australia and detected the presence of both VCG 4B and 2A as contributors of the non-defoliating pathotypes observed in Australian cotton fields [14]. In the following season (2010/2011), VCG 1A, a defoliating pathotype, was also detected for the first time. Internationally, VCG 1A has been shown to be a highly virulent pathotype [8,14]. However, typical disease presentation and similar crop losses caused by VCG 1A internationally have not been widely observed in Australia [4]. On the other hand, VCG 2A has been associated with widespread disease and yield losses in Australian cotton fields, despite there being no reports of VCG 2A causing the same damage overseas [4].

It has been suggested that the dominating presence of *V. dahliae* VCG 2A observed in Australian cotton fields may be attributed to its ability to colonise and maintain inoculum capacity on weedy hosts common to these regions [4]. Alternative hosts have the potential to act as intermittent reservoirs for *V. dahliae*, increasing inoculum levels in the field [15]. Pathogenic *V. dahliae* VCG 2A strains have been reported infecting economically important weed plant species from cotton fields overseas [16]. The survival of *V. dahliae* as an endophyte on weed plant species has been studied in Australian cotton-growing regions [17]. The endophytic infection of hosts within the cotton field would pose an additional challenge to *V. dahliae* management by potentially enhancing the carry-over of inoculum.

The green fluorescent protein (GFP) and the mCherry fluorescent protein are two widely used fluorescent proteins suitable for expressing in *V. dahliae* [18,19]. *V. dahliae* isolates tagged with GFP or mCherry have been used to understand pathogen intercellular dissemination in cotton [18], the antifungal activities of compounds [20], the infection of seed in sunflower [21], infection responses in lettuce that are resistant or susceptible to Verticillium wilt [22], and pathogenic processes in the model species *Nicotiana benthamiana* [19].

The aim of this study was to assess the suitability of using different reporter genes to tag the two predominant *V. dahliae* VCGs present in the cotton fields of Australia, thereby addressing questions relating to their virulence on cotton hosts and mechanisms of infection. One important question that needs to be addressed is whether one or both VCG 1A and VCG 2A pathotypes have the potential to endophytically colonise common weed plant species and whether differences in their capacity to colonise weeds may explain the prevalence of VCG 2A over VCG 1A in the Australian cotton field. With that in mind, the other objective was to develop an accurate screening system using fluorescence to assess the colonisation rates of *V. dahliae* on non-cotton hosts including weeds, thus shedding light on its survival over cropping cycles, allowing disease control and management to be improved. 

## 2. Materials and Methods

### 2.1. Fungal Isolates

Two isolates, *V. dahliae* strains Vd71171 (BRIP71171, Queensland Department of Agriculture and Fisheries culture collection, Queensland, Australia) and Vd71181 (BRIP71181), were used in this study, and they are henceforth referred to as VCG2A-WT (wild type) and VCG1A-WT, respectively (Table 1). 

### 2.2. Plant Materials Used in this Study

Untreated and de-linted cotton seeds without prior fungicide treatment from cultivars Siokra 1–4 and Sicot 714B3F were used in this study. Siokra 1–4 has been shown to be susceptible to the fungus (https://csd.net.au/variety-guide/; accessed on 26 February 2024), whilst Sicot 714B3F is generally considered to be tolerant to *V. dahliae*. 

Seven weed plant species collected from cotton-growing region of Narrabri (NSW, Australia) were used for testing colonisation of non-cotton hosts by *V. dahliae* in this study. They include flaxleaf fleabane (*Conyza bonariensis*), common sowthistle (*Sonchus oleraceus*), wild oats (*Avena fatua*), windmill grass (*Chloris truncata*), feathertop Rhodes grass (*Chloris virgata*), liverseed grass (*Urochloa panicoides*), and awnless barnyard grass (*Echinochloa colona*). Seeds from a native tobacco relative with known susceptibility to *V. dahliae*, *N. benthamiana*, were also used [19].

### 2.3. Vector Construction and Transformation

VCG2A-WT was previously transformed with a construct to express eGFP (ectopic expression of GFP) driven by the strong constitutive translation elongation factor 1 alpha promoter from *Aspergillus nidulans* [20]. These transformants are used to characterise Verticillium wilt in the current study.

For transforming VCG1A-WT, a vector designed to express the mCherry derivative of DsRed was obtained. Except for the mCherry coding sequence, the final vector was the same as the eGFP plasmid described previously [20]. Briefly, the cloning proceeded via the isolation of the plasmid backbone from pPZPnat1-TEF-eGFP-yeast using *Bam*HI and use of PCR products encoding the TEF promoter, fungal codon-optimised *mCherry* coding sequence (synthesised by Integrated DNA Technologies, Coralville, IA, USA) and the TEF terminator in yeast-mediated recombinatorial cloning. Primers are presented in Table S1. *Agrobacterium tumefacien* strains AGL1 and EHA105 were used to transfer the pPZPnat1-TEF-mCherry-yeast plasmid into *V. dahliae* using the method previously described [20]. Successful transformation was confirmed by plating colonies onto yeast synthetic drop-out media lacking uracil and tryptophan (Merck, Darmstadt, Germany) amended with 2% glucose. Plasmid sequence was verified by Sanger sequencing. AGL1 was grown on LB media supplemented with 50 µg/mL of both rifampicin and ampicillin, whereas EHA105 was grown without any antibiotics. 

### 2.4. Assessment of Fungal Transformants

VCG2A-WT and two eGFP-expressing transformants 71T0003 and 71T0006, henceforth referred to as VCG2A-GFP3 and VCG2A-GFP6, respectively, were inoculated on half-strength potato dextrose agar (PDA) plates of five technical replicates each strain and grown at 24 °C under a 12 h light/12 h dark photoperiod before colony diameters were measured. At 18 dpi, 10–15 mL of sterile water was added to each plate to obtain a spore suspension. Then, the spore concentration was determined using a hemocytometer. Total amount of conidia per mm^2^ was then calculated as previously described [26]. 

For the defoliating strain VCG1A-WT, four transformants, namely 81T0069, 81T0073, 81T0030, and 81T0028, were obtained and are henceforth referred to as VCG1A-mCherry69, VCG1A-mCherry73, VCG1A-mCherry30, VCG1A-mCherry28, respectively. Five technical replicates of each transformant were grown on half-strength PDA plates. Spore morphology, fluorescence, growth rates, and spore production were assessed using the Cytation 1 Cell Imaging Multi-Mode Reader (Bio Tek, Winooski, VT, USA) and Gen5 imaging software (ver 3.15.15). Briefly, a spore suspension containing 2 × 10^5^ spores/mL in PDB was aliquoted into a 96-well MicroWell™ flat plate (Thermo Fisher Scientific, Waltham, MA, USA). Bright field and mCherry fluorescence images of spores were then taken over 30 h at 1 h imaging intervals. The object sum area (µm^2^), fluorescence total intensity (RFU), and cell count were calculated for each image. Maximal growth rate (µm^2/^hr) was determined as the maximal slope along the Object Sum Area growth curve and was calculated using the Bio Tek Gen5 software (ver 3.15.15).

### 2.5. Plant Growth and Pathogenicity Assay

The non-defoliating and defoliating strains’ tests were performed over different year periods. Due to space limitations and with logistics being taken into account, we had different growth parameters for testing the different isolates. 

Siokra 1–4 and Sicot 714B3F seeds were potted in seedling trays of 35 cm × 29 cm × 5.5 cm (length, width, depth) using steam-pasteurised UQ23 mix (70% composted pine bark and 30% coco peat, University of Queensland, Brisbane, Australia). Conditions were maintained at 28 °C/24 °C (day/night, defoliating strain experiments) or 24 °C/20 °C (non-defoliating strain experiment) with a 16 h photoperiod and 64%/80% humidity until 8 days post-germination. The elevated temperature for testing the defoliating strain initially aimed to mimic the hot conditions during the Australia cotton growing season. Upon emergence of true leaves, seedlings of similar size were uprooted, and the roots were washed with water and then dipped in 500 mL of conidia suspension for 5 min at a concentration of 1 × 10^5^ (defoliating strain) and 1 × 10^6^ (non-defoliating strain) conidia/mL. A negative control was included where seedlings were root-dipped in sterile distilled water only. Plants were re-planted into round pots (140 mm in diameter) using UQ 23 mix amended with NPK fertiliser (Osmocote^®^, Marysville, OH, USA) (4 g/L). Plants were watered every 2–4 days.

For non-defoliating VCG2A-WT and VCG2A-GFP3 testing, inoculated Siokra 1–4 and Sicot 714B3F plants were moved to a temperature-controlled glasshouse set at 25 °C. Pots were placed on saucers and contained within a 23 cm × 16 cm biohazard plastic bag (Winc, Richlands, Australia) to avoid cross-contamination. Pot positions were randomised on a single bench. Four plants per pot and 12 pots per treatment (*n* = 48) were included. 

In addition to the above, 2–4 plants inoculated with VCG2A-WT and VCG2A-GFP3 were used for destructive examination and observation under a confocal microscope at 4 hpi, 1 dpi, 5 dpi, 7 dpi. Water-dipped plants were used as controls. 

Plants inoculated with the defoliating VCG1A-WT and the derivative transformants were moved back in the growth chamber and their positions were randomised. Growth conditions were adjusted to 24 °C/20 °C (16 h day/8 h night) post-inoculation. 

Upon emergence of the second true leaf, plants belonging to weed plant species common to cotton growing regions in Australia, namely *C. bonariensis* (flaxleaf fleabane), *S. oleraceus* (common sowthistle), *A. fatua* (wild oats), *C. truncata* (windmill grass), *E. colona* (awnless barnyard grass), *C. virgata* (feathertop Rhodes grass), and *U. panicoides* (liverseed grass) were root-inoculated with VCG1A-mCherry69 and VCG2A-GFP3. *N. benthamiana* (native tobacco) plants served as a *V. dahliae*-susceptible host. Plant stem tissue was surface-sterilised and then plated onto half-strength PDA at 4 weeks post-inoculation (4 wpi).

### 2.6. Scoring

**Figure 1:**
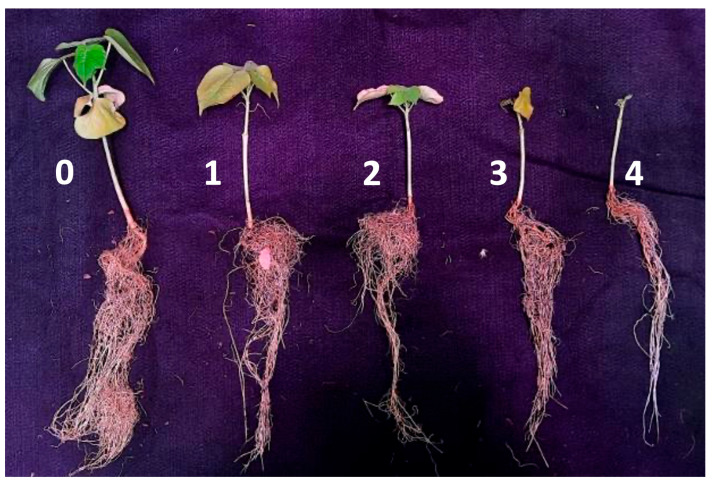
Representative cotton plants (Sicot 714B3F) challenged with a defoliating *Verticillium dahliae* strain VCG1A-WT showing disease symptoms and progression. The plants examined were taken from the same pot trial as the one described in the methods. A scale of 0 to 4 depicts the severity of necrosis, chlorosis, and wilting of leaves, as well as stunting of plant stems when compared to the uninoculated plant. A disease score of 5 indicates a dead plant.

Disease severity was scored using the following empiric scale from 0 to 5 (Table 2) [27] for the non-defoliating strains or a Likert rating scale from 0 to 5 for the defoliating *V. dahliae*-induced symptoms (Table 3) [28]. Plants were assessed for external symptoms visualised as chlorosis, necrosis, and wilting of leaves at 4 weeks post-inoculation (Figure 1). 

### 2.7. Re-Isolation

At 4 weeks post-inoculation, 10–15 cm stems from the base of the plants were destructively sampled under sterile conditions for *V. dahliae* re-isolation using a previously described method [29]. The tissues were surface-sterilised in 70% ethanol for 5 s and were blotted dry. Approximately 5 mm × 2 mm pieces were embedded into half-strength PDA containing 100 ppm streptomycin sulfate and nourseothricin 50 µg/mL (for transformants only). 

For weed plant species, cross sections of the stems were assessed for internal symptoms prior to embedding into PDA. Both transverse and longitudinal sections from stem and roots were analysed for the presence of transformed strains under a confocal microscope. Additional samples, including base of the stem, root–stem junction, leaf nodes, leaf surface, and lateral and primary root tips were also analysed under a confocal microscope.

For plants inoculated with VCG1A-WT and the mCherry transformants, single spores were further obtained from the culture of stem sections on half-strength PDA. DNA was extracted from each isolate using a rapid extraction method [30], and PCR was performed using Verticillium-specific ITS1 primers [31] to confirm their identity (Table S2).

### 2.8. Confocal Microscopy

A Zeiss 700 laser scanning microscope was used to detect the transgenic fungi with excitation at 488 nm (eGFP) and 555 nm (mCherry). eGFP and mCherry emission wavelengths were typically detected at 500–550 nm and 550–700 nm, respectively. Sections of 0.5–1 mm in diameter were excised by using a sterile razor blade from the main root, root cap, lateral roots, lateral root junctions, basal stem, and petiole (Figure S1). eGFP examinations in plants inoculated with the non-defoliating *V. dahliae* strain were repeated across two experiments. Samples were examined within two hours of sectioning to avoid autofluorescence from plant phenolic compounds or the decline of fluorescence over time. Images were captured and processed in the software ZEN Blue v3.1 (Zeiss, Oberkochen, Germany).

### 2.9. Statistical Analysis

Statistical analysis was performed in SPSS statistics for Macintosh v29 (IBM Corp, Armonk, NY, USA). Shapiro–Wilk normality test was first performed to assess whether the dataset fits a normal distribution. One-way analysis of variance (ANOVA) was then performed to derive the descriptive statistics including the means and the 95% confidence interval of each treatment group. Homogeneity of variance was also assessed using Levene’s statistics. For all except disease scores from the pathogenicity testing and weed experiments, a post hoc Tukey honestly significant difference test was performed using sample size harmonic means for unequal sample sizes to separate means for groups in homogeneous subsets.

Non-normal data including disease scores from the pathogenicity assay and weed experiments were analysed across treatment groups using the non-parametric, rank based Kruskal–Wallis H test. The threshold for statistical significance was set at a *p*-value of 0.05. 

Graphs were produced using Microsoft Excel (2024).

## 3. Results

### 3.1. In Vitro and in Plantae Assessment of VCG 2A Transformants of VCG2A-WT

**Figure 2:**
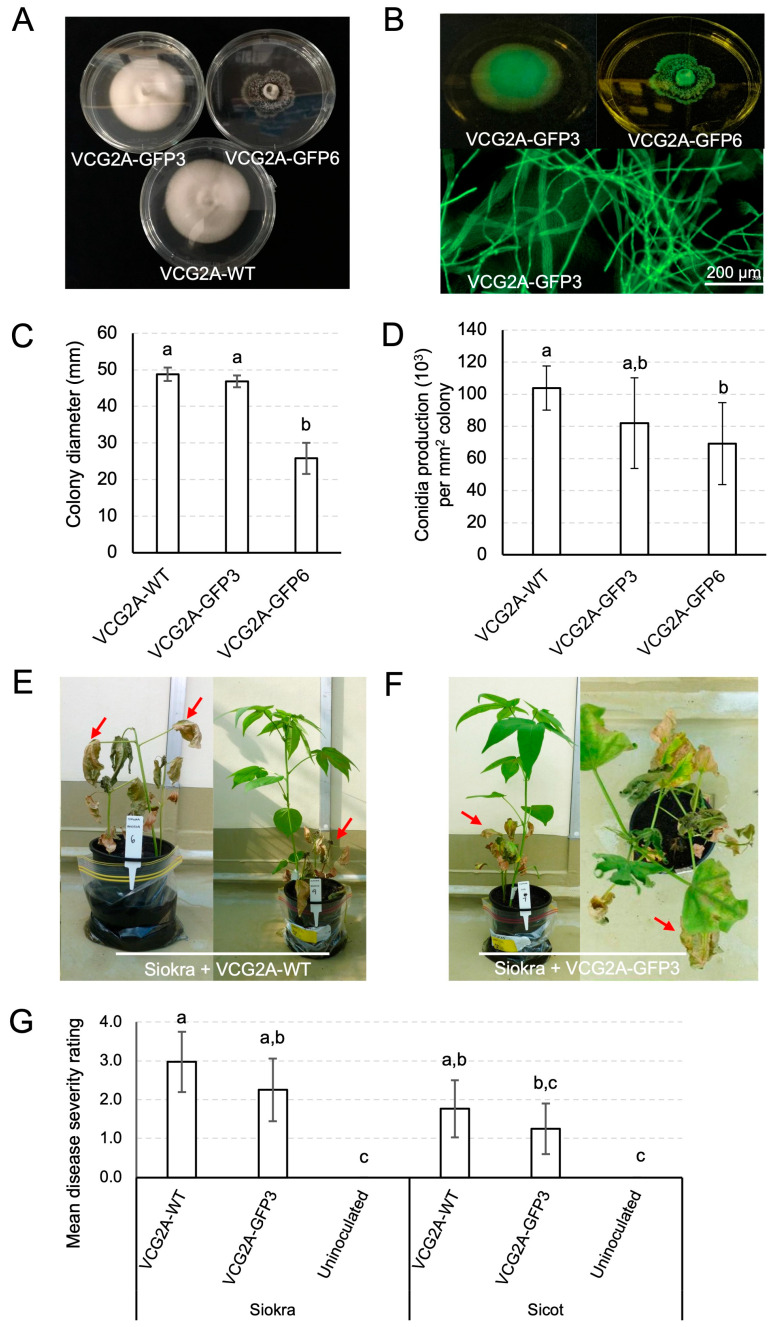
Characterisation of non-defoliating *Verticillium dahliae* isolates carrying eGFP in comparison with VCG2A-WT. (**A**) Colony morphology of *V. dahliae* wild-type strain VCG2A-WT and its eGFP transformant derivatives, VCG2A-GFP3 and VCG2A-GFP6, grown on half-strength PDA for 18 days. (**B**) Expression of eGFP on VCG2A-GFP3 and VCG2A-GFP6 colonies visualised using an ultraviolet torch (**top**). Expression of eGFP in mycelia of VCG2A-GFP3 in a magnified view (**bottom**). Scale bar = 200 µm. (**C**) Colony diameter of VCG2A-WT, VCG2A-GFP3, and VCG2A-GFP6. (**D**) Conidia production of VCG2A-WT, VCG2A-GFP3, and VCG2A-GFP6 determined after 7 days of growth on half-strength PDA. (**E**) Siokra 1–4 plants inoculated with VCG2A-WT, 4 weeks post-inoculation. Arrows indicate advanced necrosis spreading across the entire plant (**left**) and sometimes seen as severe wilting amongst healthy plants grown in the same pot (**right**). (**F**) Siokra 1–4 plants inoculated with VCG2A-GFP3, 4 weeks post-inoculation. Arrows indicate advanced necrosis on symptomatic plants (**left**) and a top-down view of a plant with necrosis spreading along the leaf margins (**right**). (**G**) Disease severity scored on Siokra 1–4 and Sicot 714B3F plants at 4 weeks post-inoculation with either VCG2A-WT or VCG2A-GFP3. Uninoculated plants served as negative controls. Letters indicate separation of means with significant differences at *p* < 0.05. Error bars indicate a 95% confidence interval. *n* = individual plants tested per isolate per cotton variety.

We previously described a transformant of *V. dahliae* that expressed strong constitutive eGFP. Of 21 stably transformed *V. dahliae* isolates, 2, VCG2A-GFP3 and VCG2A-GFP6, were selected for further characterisation based on contrasting colony morphology (Figure 2A) and the strength of eGFP fluorescence (Figure 2B). Colonies of VCG2A-WT appeared white, with raised elevation from aerial hyphae, and circular. No microsclerotia were observed when colonies were examined under a microscope. The colony of VCG2A-GFP3 appeared similar to that of VCG2A-WT (Figure 2A). Conversely, the colony of isolate VCG2A-GFP6 appeared dark with an abundance of microsclerotia when examined under a microscope and was flat in elevation, with an irregular colony margin. The colony diameter of all three isolates was measured over a period of 18 days (Figure 2C). VCG2A-GFP3 was comparable in size to VCG2A-WT, while VCG2A-GFP6 was significantly smaller in size than VCG2A-WT at *p* = 0.05. The production of conidia in water was not significantly different in VCG2A-GFP3 but was significantly reduced by 33.2% in VCG2A-GFP6 when compared to VCG2A-WT (*p* = 0.033) (Figure 2D). Therefore, VCG2A-GFP3 was subsequently used for virulence testing, as its colony morphology, growth, and conidia production conformed with those of VCG2A-WT. 

Typical Verticillium wilt symptoms, including chlorosis, necrosis, leaf yellowing, and wilting, were observed on plants inoculated with VCG2A-WT or VCG2A-GFP3 (Figure 2E,F). Internally, brown discolouration in the vasculature was observed in stem sections of diseased plants. Plants often showed varied disease severity in the same pot (Figure 2E). No external symptoms were observed on uninoculated plants. Disease severity was not statistically different between plants inoculated with VCG2A-WT and VCG2A-GFP3 (Figure 2G). For both isolates, disease severity was higher on Siokra 1–4 (average 3.0 and 2.3 for WT and VCG2A-GFP3, respectively) than Sicot 714B3F (average 1.8 and 1.3 for WT and VCG2A-GFP3, respectively). This aspect was further supported by the reisolation of these isolates at 4 wpi from diseased plants, when both isolates were re-isolated at higher frequency from Siokra 1–4 (94%, VCG2A-WT and 100%, VCG2A-GFP3) than Sicot 714B3F (39%, VCG2A-WT and 83%, VCG2A-GFP3) (Table 4).

### 3.2. Infection of Cotton Plants by V. dahliae

**Figure 3:**
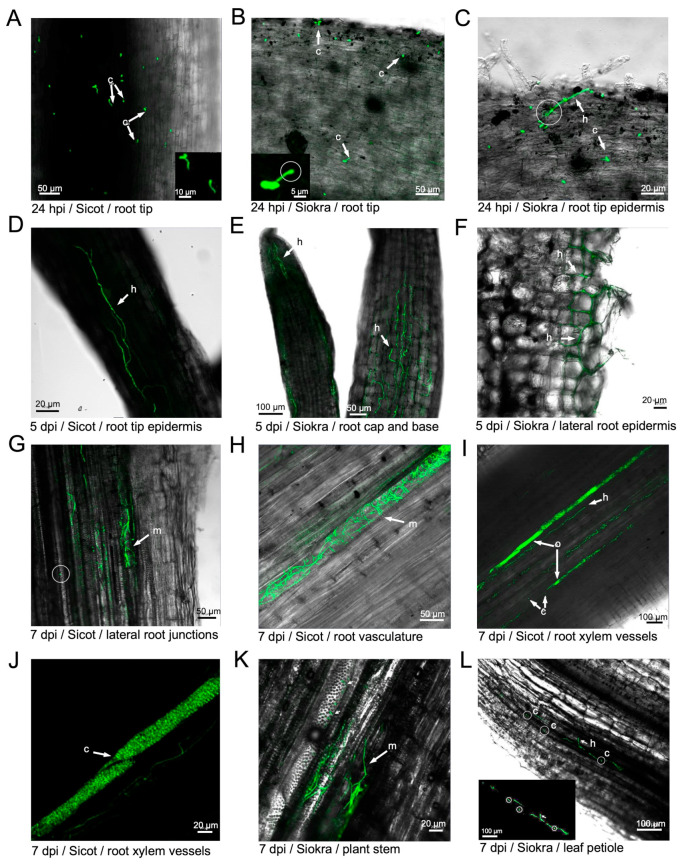
Confocal laser scanning microscopy performed at 24 hpi, 1 dpi, 5 dpi, and 7 dpi on Sicot 714B3F and Siokra 1–4 cotton cultivars inoculated with the eGFP transformant VCG2A-GFP3. (**A**) Conidia with germ tubes and hyphal elongation observed on the root tip of Sicot 714B3F at 24 hpi. Inset: magnified view of germinated conidia. (**B**) Conidia and an infection peg observed on the root tip of Siokra 1–4 at 24 hpi. Inset: magnified view of the infection peg under single-channel view. (**C**) Hyphal elongation and penetration into the root tip epidermis of Siokra 1–4 at 24 hpi. (**D**) Hyphal growth on the root tip epidermis of Sicot 714B3F at 5 dpi. (**E**) Mycelia visualised in the root cap (**left**) and at the base of the root tip (**right**) in Siokra 1–4 at 5 dpi. (**F**) Intercellular movement of hyphae through lateral root epidermis on Siokra 1–4 at 5 dpi. (**G**) Mycelia visualised in the xylem vessels of the main root in proximity to lateral root junctions in Sicot 714B3F at 7 dpi. Circled area = free-moving spore observed in the xylem. (**H**) Mycelia visualised in an entire xylem vessel of the root vasculature in Sicot 714B3F at 7 dpi. (**I**) Mycelia and conidia visualised in multiple xylem vessels of the root. Sites of vascular occlusion (o) were observed. (**J**) Single channel magnified view on the site of vascular occlusion in the xylem vessel densely packaged with conidia. (**K**) The movement of mycelia into the stem vasculature on Siokra 1–4 at 7 dpi. (**L**) Presence of mycelia and free conidia was observed in the petiole of Siokra 1–4 at 7 dpi. Inset = single channel view of the region containing mycelia and conidia (circled). Arrows show the presence of hyphae (h), mycelial networks (m), and conidia (c). Scales are indicated by horizontal bars.

To assess the colonisation process of cotton by the non-defoliating strain of *V. dahliae*, a time course of infection was performed using VCG2A-GFP3. At 4 hpi, spores were attached to the epidermis layer of Sicot 714B3F and Siokra 1–4. At 24 hpi, an abundant amount of germinated conidia was attached to the root tip epidermis in both Sicot 714B3F and Siokra 1–4 (Figure 3A), and the presence of a penetration peg (Figure 3B) and hyphal elongation (Figure 3C) were clearly observed (Table S3). At 5 dpi, hyphae were clearly observed on the root tips of both cultivars (Figure 3D,E). Mycelial networks were established along both the surface of and within the root epidermis (Figure 3E,F), with an evident intercellular movement of hyphae from the root surface towards the root cortex (Figure 3F). At 7 dpi, advanced colonisation by fungus in the vessels of lateral and main roots was observed (Figure 3G–J). New conidia produced by the fungus were also observed in these regions. In the same vessel, the proliferation of newly produced conidia appeared to occlude a section of the xylem (Figure 3I). Cells observed using a single channel at an increased magnification suggest that they are ovoid, ranging from 2.2 to 3.8 µm in length (Figure 3F). Similar observations were made on Siokra 1–4 plants (Table S3). Furthermore, mycelia were detected in the vasculature and the adjacent vessels of basal stem sections of Siokra 1–4 (Figure 3K). Hyphal tips were shown emerging through the vessel wall into an uninfected vessel. Consistent with this observation, hyphae were observed in the vessels of the petiole and appeared to have penetrated the shared vessel wall (Figure 3L). Several germinating conidia were also observed within the colonised xylem vessel. The fungus was not observed in sections of the petiole from Sicot 714B3F (Table S3).

### 3.3. Development of a Defoliating V. dahliae Strain Expressing mCherry

**Figure 4:**
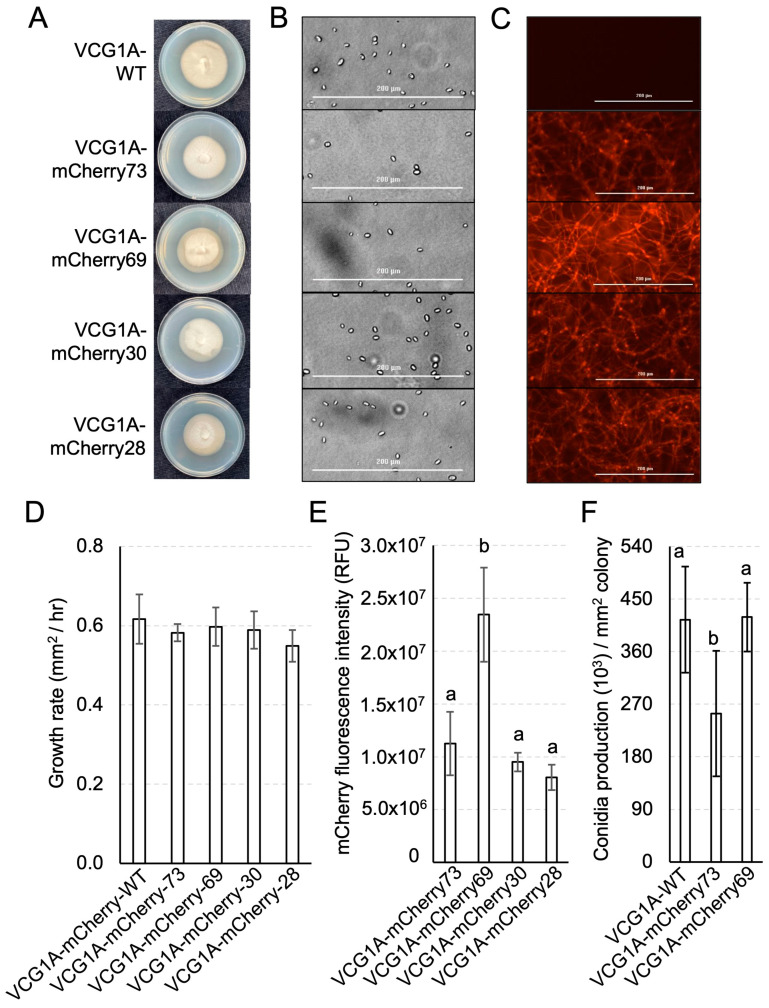
The development and visualisation of defoliating *Verticillium dahliae* strains of VCG 1A carrying the mCherry fluorescent protein. (**A**) Colonies of mCherry transformants compared to VCG1A-WT after 10 days of growth on half strength PDA. (**B**) Spores of the parent and the transformants visualised on a Bio Tek Cytation 1 imager. Scale bar = 200 µm. (**C**) Fluorescence of spores and hyphae of *V. dahliae* parent and transformant strains imaged using Bio Tek Cytation 1 Multi-Reader and Gen5 software (ver 3.15.15). Scale bar = 200 µm. (**D**) Growth rates (mm^2^/hr) of the isolates in half-strength PDB media over a 30 h period. No significant differences in growth rates were detected (*p* = 0.101, one-way ANOVA). (**E**) mCherry total fluorescence intensity (RF) of the isolates were quantified using Bio Tek Cytation 1. (**F**) Conidia production of the isolates per mm^2^ of colony after 10 days of growth on half-strength PDA media. Error bars show a 95% confidence interval. Statistics performed with a negative binomial generalised linear model. (**D**–**F**): Letters indicate separation of means with significant differences (*p* < 0.005) detected between groups using one-way ANOVA followed by post hoc Tukey test.

Regarding the *V. dahliae* isolates obtained from VCG1A-WT, 53 fluorescent transformants were obtained after *A. tumefaciens* transformation. A total of 36 of these were isolated from AGL1 of A. tumefaciens, while 17 were obtained from *A. tumefaciens* strain EHA105. Of these, 4 isolates, VCG1A-mCherry28, 30, 69, and 73, were selected for further analysis based on fluorescence level and morphological features (Table S4, Figure 4A–C). The macroscopic morphology of the four transformants was consistent with that of VCG1A-WT (Figure 4A). Both VCG1A-WT and transformant isolates produced hyaline spores, cylindrical to ovate in shape, and approximately 5 µm in length (Figure 4B). No significant differences (*p* = 0.101) in terms of growth rate were observed between VCG1A-WT and the transformants (Figure 4D). The quantification of total mCherry fluorescence in each transformant shows that a higher intensity (*p* < 0.001) was observed in VCG1A-mCherry69 than the rest (Figure 4E). No significant difference (*p* = 0.821) in conidia production was observed between VCG1A-WT and VCG1A-mCherry69 (Figure 4F). Conversely, VCG1A-mCherry73 had a lower conidia production than VCG1A-WT and VCG1A-mCherry69 (*p* < 0.05). Therefore, based on the intensity of the fluorescence, uniformity in growth rate, and spore production relative to VCG1A-WT, VCG1A-mCherry69 was carried forward for the further testing of its virulence on cotton plants.

**Figure 5:**
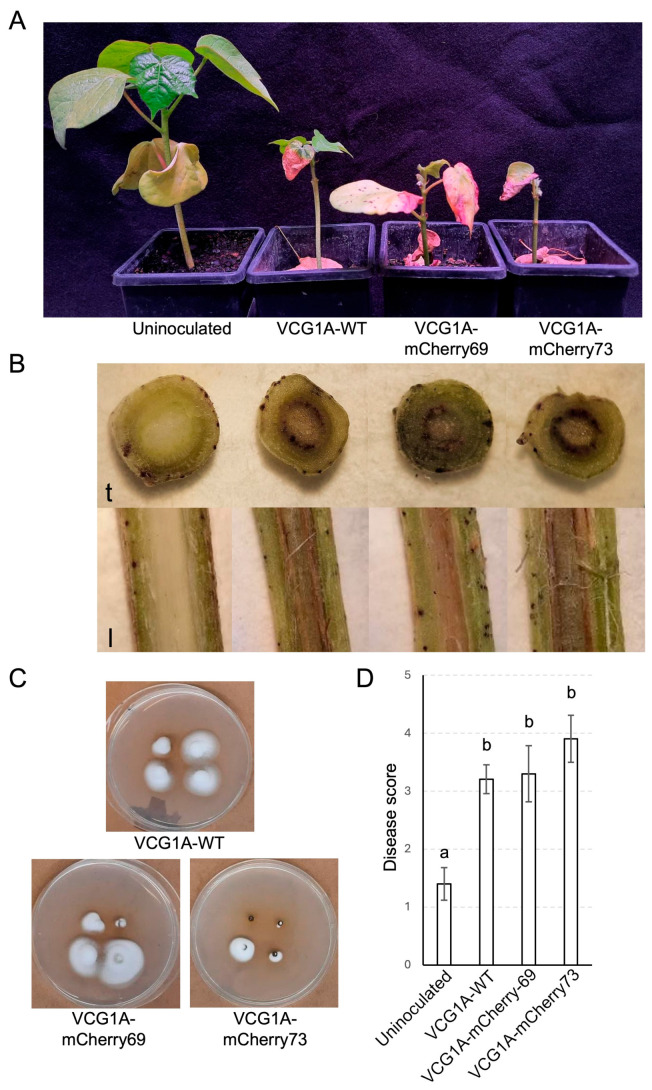
Pathogenicity assay of *Verticillium dahliae* VCG1A-WT and transformants VCG1A-mCherry69 and VCG1A-mCherry73 on Sicot 714B3F. (**A**) Assessment of symptoms in Sicot 714B3F seedlings 4 weeks post-inoculation. Plants were rated according to Cirulli et al. (1990) and were scored as the following: one for the uninoculated plant, three for VCG1A-WT inoculated plant, three for a VCG1A-mCherry69 inoculated plant, and four for a VCG1A-mCherry73 inoculated plant. (**B**) Transverse (t) and longitudinal (l) stem sections of symptomatic plants showing visible discolouration in the vasculature. (**C**) *V. dahliae* colonies recovered from sections of the stem tissues of symptomatic Sicot 714B3F seedlings after 10 days of incubation on half-strength PDA plates. Individual plates show four samples taken from one symptomatic seedling. (**D**) Mean disease scores in Sicot 714B3F plants inoculated with VCG1A-WT (*n* = 24), VCG1A-mCherry69 (*n* = 20), and VCG1A-mCherry73 (*n* = 21). Sterile distilled water was used as the uninoculated control. Error bars indicate a 95% confidence interval. Letters indicate the separation of means between the isolates at *p* < 0.05.

Sicot 714B3F seedlings root-inoculated with VCG1A-WT, VCG1A-mCherry69 and VCG1A-mCherry73 at 4 wpi showed disease severity that was significantly higher (*p* < 0.001) than the uninoculated controls. Internal red-brown discolouration was observed in the stem vasculature of plants inoculated with the transformants or VCG1A-WT while they were absent in the uninoculated plants (Figure 5B). *Verticillium dahlie*-like colonies were reisolated from surface-sterilised plant tissues on half-strength PDA plates (Figure 5C), and the identification at the species level was confirmed by the analysis of ITS sequences on VCG1A-WT (Table S2) and the detection of mCherry fluorescence in the transformant isolates. The parent Vd71181 strain was reisolated from 62.5% (*n* = 24) of inoculated seedlings. VCG1A-WT was reisolated from 62.5% of inoculated seedlings, while VCG1A-mCherry69 and VCG1A-mCherry73 were reisolated from 45.0% and 66.7% of the inoculated plants, respectively. *Verticillium dahliae* was not reisolated from uninoculated seedlings. All *V. dahliae*-inoculated plants showed a significantly higher disease severity (*p* < 0.001) compared to the uninoculated plants (Figure 5D). There was no significant difference (*p* = 0.076) in disease severity between seedlings inoculated with VCG1A-WT (average, 3.2) and the transformants VCG1A-mCherry69 (average 3.3) and VCG1A-mCherry73 (average 3.9) (Figure 5D).

**Figure 6:**
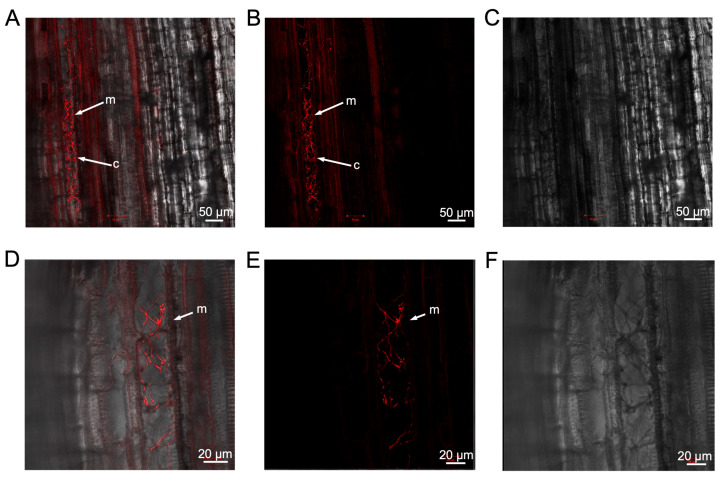
Localisation of *Verticillium dahliae* in Sicot 714B3F cotton seedlings inoculated with the mCherry expressing transformant, VCG1A-mCherry69 at 28 days post-inoculation. (**A**) Confocal microscopy image of longitudinal stem section of Sicot 714B3F showing the colonisation of host xylem tissues by mCherry-expressing mycelia. mCherry fluorescence is visualised with an overlay of plant tissue in T-PMT transmission illumination mode. (**B**) mCherry fluorescence visualised in single channel only. (**C**) T-PMT mode only showing the bright field of plant structure, without the laser scanning mode. (**D**) Magnified view of the cortex region near the xylem and the proliferation of mCherry-tagged mycelia in this region. (**E**) mCherry fluorescence in the cortex region visualised in single channel. (**F**) T-PMT mode only showing the bright field of cortex region in the stem. Bars indicate the scale used to capture each image. Arrows show the presence of mycelial networks (m) and conidia (c). Viewed at magnification 10× using EC Plan-Neofluar objective. Laser excitation = 555 nm, Master Gain = 809.

To determine if the defoliating strain of *V. dahliae* colonised cotton plants in a manner similar to the non-defoliating strain, longitudinal sections of the stem of Sicot 714B3F inoculated with VCG1A-mCherry69 were dissected 28 days after inoculation and visualised under a confocal microscope. mCherry-tagged mycelia were clearly present in the xylem tissues of Sicot 714B3F plants (Figure 6A–C) and colonising the root cortex (Figure 6A). A magnified view suggested that the mycelia were within the parenchyma cells adjacent to the xylem in this region (Figure 6D–F). Although a side-by-side comparison has not been made, the colonisation process appeared similar for both the defoliating and non-defoliating strains.

### 3.4. Both Defoliating and Non-Defoliating V. dahliae Can Colonise Weed Species and Cause Disease in Nicotiana benthamiana

**Figure 7:**
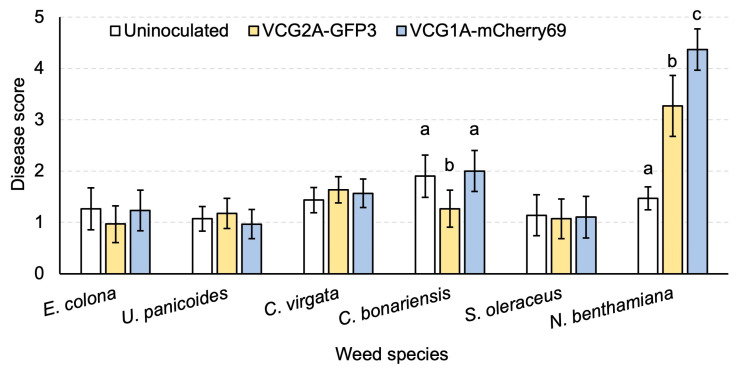
Disease severity of *Verticillium dahliae* transformants VCG2A-GFP3 and VCG1A-mCherry69 on weed plant species and *Nicotiana benthamiana*. Comparison of mean disease scores in weed plant species and *N. benthamiana* 4 weeks after inoculation. Letters indicate separation of means amongst treatment groups for *Conyza bonariensis* and *N. benthamiana* at *p* < 0.05. There was no significant difference amongst the other treatment groups. Error bars represent a 95% confidence interval.

*V. dahliae* transformant strains of VCG1A-mCherry69 and VCG2A-GFP3 were used to inoculate seven common weed plant species commonly found in Australian fields. At 4 wpi, plants were visually examined (Figure S2). There were no noticeable differences in the size of the plants, the colour of the leaves, or stem height between VCG2A-GFP3 and VCG1A-mCherry69 inoculated weeds and their respective uninoculated controls (Figure S2). This was also reflected in terms of disease severity (Figure 7). All weed plant species showed minimal symptoms, indicating that they are tolerant to these *V. dahliae* strains. *C. bonariensis* showed slightly elevated leaf yellowing on uninoculated plants and on plants inoculated with VCG1A-mCherry69 (Figure 7). Conversely, significantly elevated disease severity was detected (*p* < 0.05) in inoculated versus uninoculated *N. benthamiana* plants (Figure 7). The impact of the inoculum on *N. benthamiana* was evident, with severe stunting observed on tobacco plants inoculated with either VCG2A-GFP3 or VCG1A-mCherry69 (Figure S2H).

**Figure 8:**
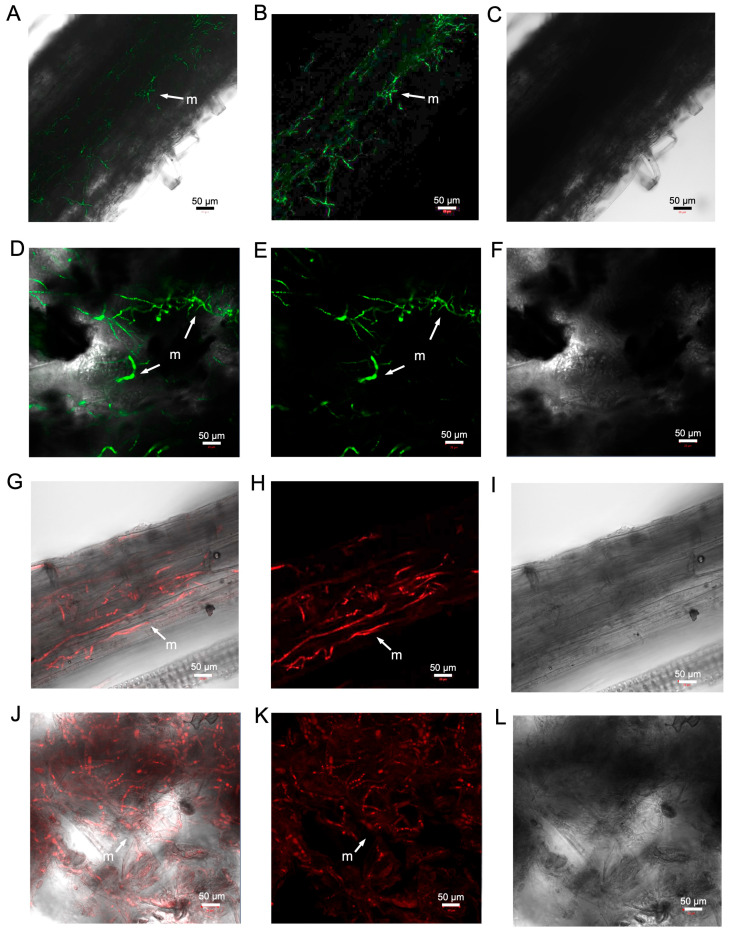
Confocal micrographs visualised on *Nicotiana benthamiana* challenged with VCG1A-mCherry69 and VCG2A-GFP3 at 4 weeks post-inoculation. (**A**) Mycelial networks of VCG2A-GFP3 visualised on a section of the base of a stem. (**B**) eGFP fluorescence of VCG2A-GFP3 in the stem visualised on a single channel. (**C**) T-PMT mode only showing the bright field of VCG2A-GFP3 in the stem. (**D**) Mycelial networks of VCG2A-GFP3 visualised on a section of a leaf. (**E**) eGFP fluorescence of VCG2A-GFP3 in the leaf visualised on a single channel. (**F**) T-PMT mode only showing the bright field of VCG2A-GFP3 in the leaf. (**G**) mCherry fluorescence of VCG1A-mCherry69 visualised on a section of a root. (**H**) mCherry fluorescence of VCG1A-mCherry69 in the root visualised on a single channel. (**I**) T-PMT mode only showing the bright field of VCG1A-mCherry69 in the roots. (**J**) mCherry fluorescence of VCG1A-mCherry69 visualised on a section of a leaf. (**K**) mCherry fluorescence of VCG1A-mCherry69 in the leaf visualised on a single channel. (**L**) T-PMT mode only showing the bright field of VCG1A-mCherry69 in the leaf. Laser excitation = 555 nm (mCherry), 488 nm (eGFP). Arrows indicate the presence of mycelial networks (m). Bars indicate the scale used to capture each image.

Samples including the base of the stem, root–stem junction, leaf nodes, leaf surface, and lateral and primary root tips were taken from inoculated plants of each weed species and *N. benthamiana* at 4 wpi and then analysed under a confocal microscope. Mycelial networks were detected at the base of a stem of a tobacco plant inoculated with VCG2A-GFP3 (Figure 8A–C). Further up the plant, mycelia carrying eGFP fluorescence were detected in the leaves of a tobacco plant inoculated with VCG2A-GFP3 (Figure 8D–F). Similarly, mycelial networks carrying mCherry fluorescence were detected in the roots of a tobacco plant inoculated with VCG1A-mCherry69 (Figure 8G–I). mCherry-tagged mycelial networks were also detected in the leaf of a tobacco plant inoculated with VCG1A-mCherry69 (Figure 8J–L). Weed plants inoculated with VCG2A-GFP3 and VCG1A-mCherry69 were dissected and then examined in the same manner as tobacco plants. No mycelial networks were observed on all weed tissues examined. Germinating hyphae carrying mCherry fluoresence were observed on the root epidermis of *U. panicoides* (Figure S3). This was the only instance where the presence of fluorescence-tagged fungus was detected *in plantae* in the weeds.

Defoliating and non-defoliating *V. dahliae* transformants were reisolated from three and five weed plant species, respectively (Table 5). Fungal colonies were recovered from *C. bonariensis*, *S. oleraceus*, *C. virgata*, *E. colona*, *U. panicoides,* and *N. benthamiana* (Figure S4). Individual colonies were then confirmed to be either VCG2A-GFP3 or VCG1A-mCherry69 by fluorescence under a confocal microscope (Figure S5). *V. dahliae* was not reisolated from uninoculated plants. The recovery rates of transformant VCG2A-GFP3 and VCG1A-mCherry69 across all weeds (*n* = 190) were 17.4% and 8.9%, respectively (Table 5). The highest recovery percentage of the non-defoliating VCG2A-GFP3 within weed plant species was 24% (*n* = 25) from *U. panicoides* belonging to the *Poaceae* family and 15.4% (*n* = 26) from *C. bonariensis* L. belonging to the *Asteraceae* family. The recovery rate of VCG1A-mCherry69 from the weeds was relatively low when compared to that of VCG2A-GFP3, with a 7.7% recovery rate of the fungus from *E. colona* plants (*Poaceae*) being the highest (Table 5).

Recovery rates of VCG2A-GFP3 (61.5%) and VCG1A-mCherry69 (50%) from stem sections of *N. bethamiana* were much higher than that for the weed plant species. These observations suggest that *N. benthamiana* is a susceptible plant host for *V. dahliae*.

## 4. Discussion

Verticillium wilt is a major disease for the Australian cotton industry. In Australia, it is generally considered that the non-defoliating pathotypes of VCG 4B and the more recently detected VCG 2A are the prevalent disease-causing strains in cotton fields [4,14]. Recent field incidences of Verticillium wilt within the last decade have been low but were observed to be rising steadily in successive seasons. This increased occurrence and the detection of VCG 1A pathotype from the NSW DPI culture collection [14] has raised some concerns about the cause of increased disease severity in the field. It is known that VCG 2A can infect weed plant species prevalently found in cotton fields including *S. oleraceus* that was characterised in this study [16]. Whether or not its adaptation to survive on other plant species is what makes it the dominant pathotype in Australia is not clearly understood. However, the defoliating VCG 1A is not so widespread in Australia as it has been overseas in causing crop losses and the complete defoliation of infected cotton plants [4]. This study addresses the pathogenicity of these two VCGs and paves the way for the evolution of these populations to be dissected and to aid in the practical management of this disease in the Australian cotton industry.

The use of reporter protein-tagged *V. dahliae* strains has not only allowed the pathogen to be tracked within the plant but also facilitated the recovery of the pathogens from various hosts, including those that are non-symptomatic. The eGFP-expressing VCG 2A and mCherry-expressing VCG 1A inoculated plants showed similar levels of disease severity when compared to their respective wildtype isolates, indicating that the transformation did not alter the virulence of these strains on cotton. Future work will include co-inoculation experiments to test, if any, interactions between VCG 1A and 2A.

The localisation of the eGFP-expressing VCG 2A was visualised in Siokra 1–4 and Sicot 714B3F cotton plants during a period of 7 days post-inoculation to study the early infection process. Conidia were observed on the root tips of both cultivars at 4 hpi. In another study, conidia germination was observed as early as 2 hpi on cotton [32]. At 24 hpi, germ tubes were visible on approximately 50% of conidia observed on both cultivars. This is comparable to the germination timing of an eGFP-expressing *V. dahliae* on lettuce, first observed at 12 to 48 h following inoculation [22]. An infection peg was observed on the surface of the root tip at 24 dpi. Hyphal swelling was also evident. While infection structures of *V. dahliae* in the form of appressoria were observed in penetrating the root surface of lettuce at 48 hpi and fiber flax at 1 wpi [22,33], it has not been observed in other plant species such as *N. benthamiana* [19]. *V. dahliae* showed only slight hyphal swelling without a penetration peg observed before infection in oilseed rape and sunflower [21,34]. However, a cotton-derived *V. dahliae* isolate showed slight hyphal swelling, followed by a penetration peg on Arabidopsis roots [35]. This appears to be required for the isolate to breach the cell wall of cotton root epidermis during the initial colonisation [36]. This is consistent with our observations in this study.

The root tip was colonised by the fungus, and its intercellular movement through the vascular tissues was evident at 5 dpi. This confirmed that the mechanism of *Verticillium* spp. infection is through establishing the successful colonisation of the vascular tissues, particularly the xylem elements [32,37]. This also confirmed that root tips are sites of penetration for *V. dahliae* on cotton hosts [32,38].

At 7 dpi, advanced mycelia and mycelial networks were observed. *V. dahliae* mycelia were mostly confined to the individual xylem vessels of the vasculature, with longitudinal movement in the xylem and the perforating tracheary elements. The colonisation of lateral root junctions was observed, as reported in a previous study [32]. Conidia and mycelia were detected in the stem and petiole of Siokra 1–4 but not in Sicot 714B3F. Above-ground colonisation by *V. dahliae*, specifically in the petiole base, has been previously reported [32], although it was detected at 30 days post-inoculation using a virulent non-defoliating eGFP-expressing isolate of *V. dahliae*. At 7 dpi, an intense fluorescence signal in the xylem of Sicot 714B3F was identified as a vascular occlusion caused by the proliferation of the fungus confined to the xylem vessel. Vascular occlusions by fungal pathogens are often associated with the formation of plant structures such as tyloses to inhibit the movement of the fungus inside the host. Such occlusions were typically observed as densely clustered conidia in the tracheid of oilseed rape [34]. However, sometimes, occlusions can lead to the blockage of xylem vessels and instigate the classic wilt symptoms [38]. Restricting xylem vessels colonised by *V. dahliae* in the lateral roots was identified as an important response in wilt-resistant lettuce cultivars [22]. Similarly, the cotton interaction observed here could point toward the restriction of the fungus at the border pit membranes of the xylem [8].

Typical defoliating symptoms were induced on cotton by the mCherry-expressing VCG 1A isolate. The infection process through the vasculature appears similar to the non-defoliating strain. In another study, both defoliating and non-defoliating isolates recovered from the same stem showed comparable levels of virulence when cotton plants were inoculated with either or both isolates in a pot trial [29]. Nevertheless, the detection of a defoliating pathotype even at a relatively low frequency compared to the non-defoliating pathotype complicates the landscape for disease management in the Australian cotton fields [29].

Mycelial networks with mCherry fluorescence were clearly visualised in the xylem vessels of plants inoculated with the defoliating strain. This is consistent with the brown discolourations observed in the vascular regions of symptomatic stems. Interestingly, the movement of mycelia was observed in the cortex region. However, the pattern of colonisation suggests that it was moving along the surface or in between cell layers of the cortex and endosperm. Unlike other hosts, intracellular colonisation was observed rarely in *V. dahliae* localisation studies on cotton and Arabidopsis [32,35]. Both the intra- and intercellular movement of hyphae through the endoderm were proposed [39]. However, the movement from cortical cells into xylem vessels was observed in an intercellular manner [35]. 

The emergence and widespread prevalence of the Australian VCG 2A strain has prompted investigation into the capacity for Australian *V. dahliae* isolates to colonise common Australian hosts. Previous reports propose that VCG 2A may become predominant in Australian cotton fields through its ability to infect weed plant species [4]. In this study, no statistical differences were observed in terms of reisolation frequencies from different weeds tested between *V. dahliae* VCG2A-GFP3 and VCG1A-mCherry69. However, for VCG2A-GFP3 on some weed plant species, the reisolation frequencies were higher than VCG1A-mCherry69, addressing the need to perform additional analysis with more weed plant species and replicates. 

*V. dahliae* was generally isolated from six of the eight species investigated, including the known host, *N. benthamiana* [19]. *N. benthamiana*, a native Australian plant, has been used as a model species to understand *V. dahliae* infection in previous studies [19]. However, there is limited literature describing *N. benthamiana* susceptibilities to different *V. dahliae* VCGs. Here, transformants VCG1A-mCherry69 and VCG2A-GFP3 were isolated from *N. benthamiana* plants with similar frequencies, although VCG1A-mCherry69 induced higher disease severity than VCG2A-GFP3. These findings therefore provide a deeper insight into *V. dahliae* interactions with the model species *N. benthamiana*.

Confocal microscopy did not detect the *in plantae* proliferation of both transformants in all weed plant species tested, even though one or both transformants were reisolated from the stems of five weed plant species at low frequencies. This suggests that the *V. dahliae* strains tested in this study have limited capacity to colonise these weed plant species. However, a minimal presence of the transformants was still detected in the stems of inoculated plants including *C. virgata*, *U. panicoides*, *S. oleraceus*, *C. bonariensis,* and *E. colona*. This is consistent with the roles of some of these weed plant species in acting as endophytic or susceptible hosts for *V. dahliae* [16,17,40,41,42,43,44]. 

*V. dahliae* VCG2A-GFP3 and VCG1A-mCherry69 were both reisolated from *C. bonariensis*. Some of the *C. bonariensis* seedlings were exhibiting signs of stress throughout the duration of the experiment. Tissue reisolation did not detect the presence of *V. dahliae* in the uninoculated plants. However, there was still a minimal presence of *V. dahliae* in the reisolated tissues of plants inoculated with VCG1A-mCherry, which may still explain the cause of leaf yellowing observed on these plants.

It is important to note that whilst this study identifies weed plant species that are potential carriers of *V. dahliae* VCGs 1A and 2A, it does not investigate the capacity of these hosts to increase pathogen inoculum levels in the field. *V. dahliae* microsclerotia are the primary fungal propagules that persist in soils and act as carry-over inoculum into subsequent cropping seasons [45]. Consequently, further investigations into the capacity of local weed plant species to increase the inoculum load of *V. dahliae* will help to shape future Verticillium wilt management approaches.

## 5. Conclusions

Verticillium wilt is continuing to have a major impact on crop production around the world. Specific pathotypes of *V. dahliae* have contributed to disease outbreaks in the cotton fields in Australia. The focus of this study was to highlight the importance of eGFP and mCherry genetic tools to better study the Verticillium–cotton pathosystem as well as the biological aspects of *V. dahliae* infection on weed plant species.

## Figures and Tables

**Table 1:** Australian *Verticillium dahliae* isolates obtained from *Gossypium hirsutum* (Upland cotton).

Isolate	Accession	VCG	Pathotype	Locality
*V. dahliae “Vd71171”*	BRIP71171	2A ^1^	Non-defoliating	Namoi Valley, NSW, Australia
*V. dahliae “Vd71181”*	BRIP71181	1A ^2^	Defoliating	Gwydir Valley, NSW, Australia

^1^ Vd71171 was originally isolated from the Namoi Valley, NSW, Australia and has been confirmed to group with the non-defoliating VCG 2A by nitrate-non-utilizing (*nit*) mutant complementation tests. ^2^ Vd71181 originated from Gwydir Valley, NSW, Australia and shared 100% sequence identity to *V. dahliae* through ITS sequencing (Table S2). Both isolates have been further confirmed by PCR using primers that can distinguish one pathotype from the other [23,24], while whole-genome SNP profiling has confirmed the phylogenetic position of Vd71181 within the VCG 1A subclade [25].

**Table 2:** Rating scale used to assess disease severity of cotton plants inoculated with a non-defoliating strain of *Verticillium dahliae*.

Score	Description of Symptoms
0	Healthy, no symptoms
1	1–20% total leaf area affected
2	21–40% total leaf area affected
3	41–60% total leaf area affected
4	61–80% total leaf area affected
5	81–100% total leaf area affected, and or plant death

**Table 3:** Rating scale used to assess disease severity of cotton plants, weeds, and tobacco plants inoculated with a non-defoliating strain of *Verticillium dahliae*. This scoring system is adapted from a previous study [28].

Symptoms	Affected Leaves (%)	Degree of Stunting Compared to Control ^1^		
		None or Very Slight (<10%)	Moderate (11–50%)	Severe (>50%)
None	0	0	-	-
Slight leaf chlorosis, flaccidity, necrosis	1–10	1	2	3
Moderate leaf chlorosis, flaccidity, necrosis, slight defoliation		2	3	4
	11–25			
Severe leaf chlorosis, flaccidity, necrosis, moderate defoliation	26–50	3	4	4
Plants with severe or complete defoliation	>50	4	4	4
Dead plants	–	5	5	5

^1^ Scored based on the percentage in height reduced when compared to the uninoculated control plants.

**Table 4:** Reisolation percentage of *Verticillium dahliae* isolates VCG2A-GFP3 and VCG2A-WT at 4 wpi.

	Siokra 1–4	Siokra 1–4	Sicot 714B3F	Sicot 714B3F
Treatment	Recovery/Total Diseased ^1^	Recovery/Total Symptomless ^2^	Recovery/Total Diseased ^1^	Recovery/Total Symptomless ^2^
Uninoculated	0/0	0/48 (0%)	0/0	0/48 (0%)
VCG2A-WT ^3^	31/33 (94%)	1/15 (7%)	20/24 (83%)	0/24 (0%)
VCG2A-GFP3 ^3^	24/24 (100%)	1/24 (4%)	7/18 (39%)	1/30 (3%)

^1^ Diseased plants were determined based on *V. dahliae* symptoms such as wilting, leaf chlorosis, and necrosis. ^2^ Plants inoculated with *V. dahliae* with a disease severity score of 0 were considered symptomless. ^3^ Isolates recovered were confirmed to be positives by comparing their colony morphology to VCG2A-WT and by detecting eGFP fluorescence (VCG2A-GFP3). Percentage (%) recovery is expressed as the number of plants from which the isolate was recovered from over the total number of diseased or symptomless plants.

**Table 5:** Reisolation of *Verticillium dahliae* VCG2A-GFP3 and VCG1A-mCherry69 from stem tissue of weed plant species at 4 wpi. Colony identities were confirmed on the basis of green or red fluorescence.

Family	Weed Plant Species	Isolate	Frequency ^1^
*Asteraceae*	*Conyza bonariensis* L.	VCG2A-GFP3	4/26 (15.4%)
		VCG1A-mCherry69	1/26 (3.8%)
	*Sonchus oleraceus* L.	VCG2A-GFP3	2/25 (8%)
		VCG1A-mCherry69	0/25 (0%)
*Poaceae*	*Avena fatua* L.	VCG2A-GFP3	0/10 (0%)
		VCG1A-mCherry69	0/10 (0%)
	*Chloris truncata* R.Br.	VCG2A-GFP3	0/26 (0%)
		VCG1A-mCherry69	0/26 (0%)
	*Chloris virgata* Sw.	VCG2A-GFP3	3/26 (11.5%)
		VCG1A-mCherry69	0/26 (0%)
	*Echinochloa colona* L.	VCG2A-GFP3	2/26 (7.7%)
		VCG1A-mCherry69	2/26 (7.7%)
	*Urochloa panicoides* P.Beauv.	VCG2A-GFP3 VCG1A-mCherry69	6/25 (24%) 1/25 (4%)
*Solanaceae*	*Nicotiana benthamiana* Domin	VCG2A-GFP3 VCG1A-mCherry69	16/26 (61.5%) 13/26 (50%)

*V. dahliae* was not isolated from any of the water only control plants. ^1^ Number of plants from which *V. dahliae* was isolated/total plants inoculated.

## Data Availability

The original contributions presented in the study are included in the article/Supplementary Material; further inquiries can be directed to the corresponding author/s.

## Supplementary Materials

The following supporting information can be downloaded at https://www.mdpi.com/article/10.3390/pathogens13060442/s1. Figure S1: Anatomy of a cotton seedling approximately 10–14 days after sowing; Figure S2: Assessing different weed plant species and *Nicotiana benthamiana* for their potentials to house non-defoliating (VCG 1A) and defoliating (VCG 2A) transformant strains; Figure S3: VCG1A-mCherry69 visualised on the roots of *Urochloa panicoides* at 4 weeks post-inoculation; Figure S4; Colonies reisolated from stem sections of weed plant species inoculated with VCG2A-GFP3 and VCG1A-mCherry69. Figure S5: Colonies from stem reisolations confirmed under a confocal microscope to carry GFP or mCherry proteins. Table S1: Primers for yeast recombination-based cloning to generate a plasmid for the expression of mCherry in *Verticillium dahliae*; Table S2: *Verticillium dahliae*-specific primers [31] amplifying a 200 bp ITS product were used to confirm its identity; Table S3: Summary of rate of colonisation based on timing of initial observation at each infection stage throughout the confocal microscopy experiment; Table S4: mCherry transformant isolates selected for comparison against *Verticillium dahliae* VCG 1A parent, Vd71181, that originated from Gwydir Valley, NSW.
