# Supplementary material for: Fluorescently Tagged Verticillium dahliae to Understand the Infection Process on Cotton (Gossypium hirsutum) and Weed Plant Species
Source: Pathogens. 2024 May 23;13(6):442. doi: 10.3390/pathogens13060442 (PMC11207081; doi:10.3390/pathogens13060442)
Supplement: Supplementary file 1 [file pathogens-13-00442-s001.zip › pathogens-2983062-supplementary.pdf]

pathogens

Article

Fluorescently Tagged *Verticillium dahliae* to Understand the Infection Process on Cotton (*Gossypium hirsutum*) and Weed Plant Species

Andrew Chen [1,*,†], Sabrina Morrison [1,2,†], Aphrika Gregson [1,3,†], Duy P. Le [4], Andrew S. Urquhart [5,‡], Linda J. Smith [6], Elizabeth A. B. Aitken [1,*] and Donald M. Gardiner [2,*].

Supplementary Materials

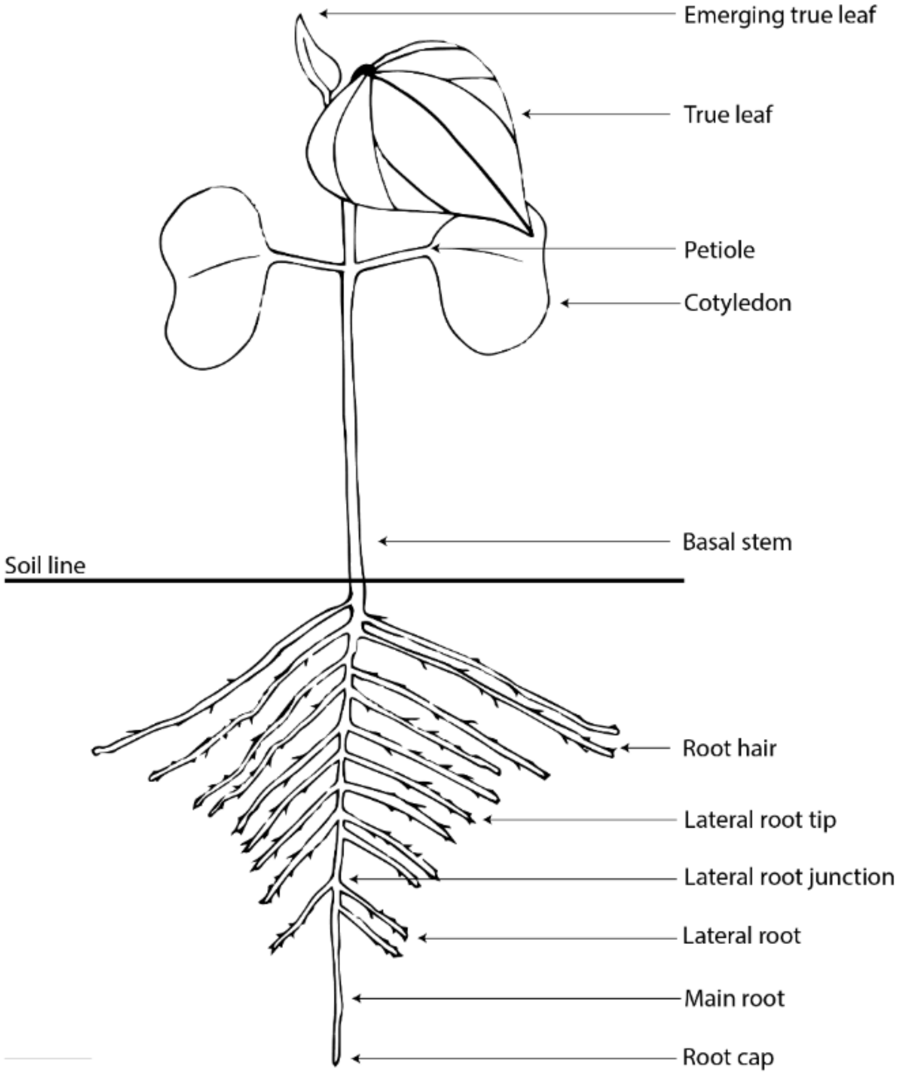

Figure S1. Anatomy of a cotton seedling approximately 10 -14 days after sowing. Plant sections excised by hand for confocal microscopy include root cap, main root, lateral root, lateral root junction and tip, basal stem, and petiole of true leaves.

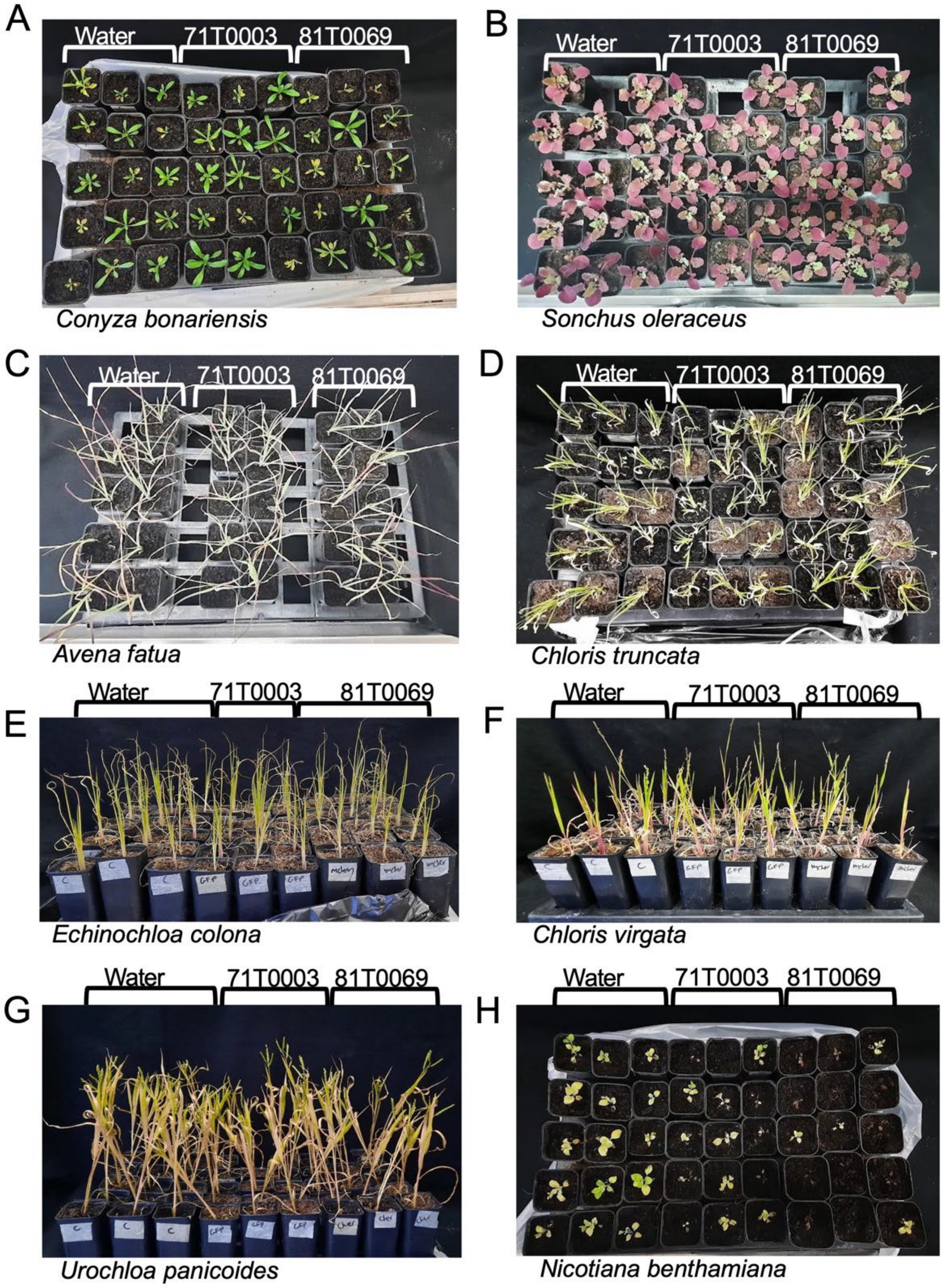

Figure S2. Assessing different weed plant species and *Nicotiana benthamiana* for their potentials to house non-defoliating (VCG 1A) and defoliating (VCG 2A) transformant strains. **(A)** *Conyza bonariensis* plants. **(B)** *Sonchus oleraceus* plants. **(C)** *Avena fatua* plants. **(D)** *Chloris truncata* plants. **(E)** *Echinochloa colona*. **(F)** *Chloris virgata* plants. **(G)** *Urochloa panicoides* plants. **(H)** *Nicotiana benthamiana* plants. *N* = 10 to 25 individual plants per treatment group.

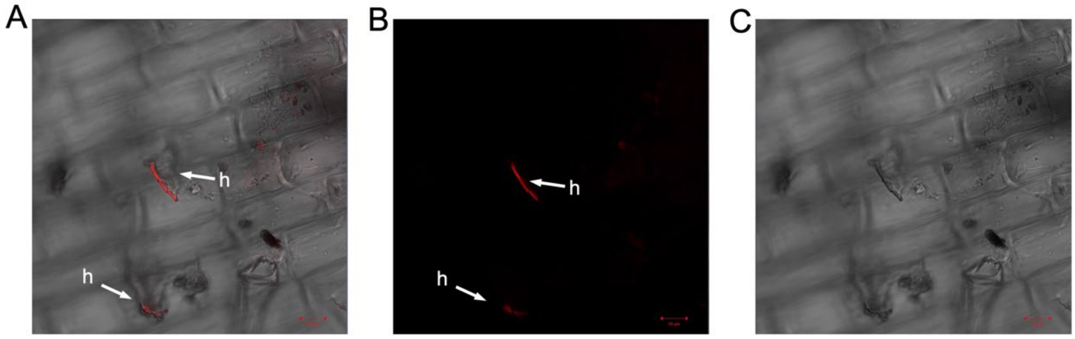

Figure S3. VCG1A-mCherry-69 visualised on the roots of *Urochloa panicoides* at 4 weeks post inoculation. **(A)** Germinating hyphae was observed on the root epidermis. **(B)** mCherry fluorescence visualised in single channel using the laser scanning mode. **(C)** T-PMT mode only showing the bright field of plant structure, without the laser scanning mode.

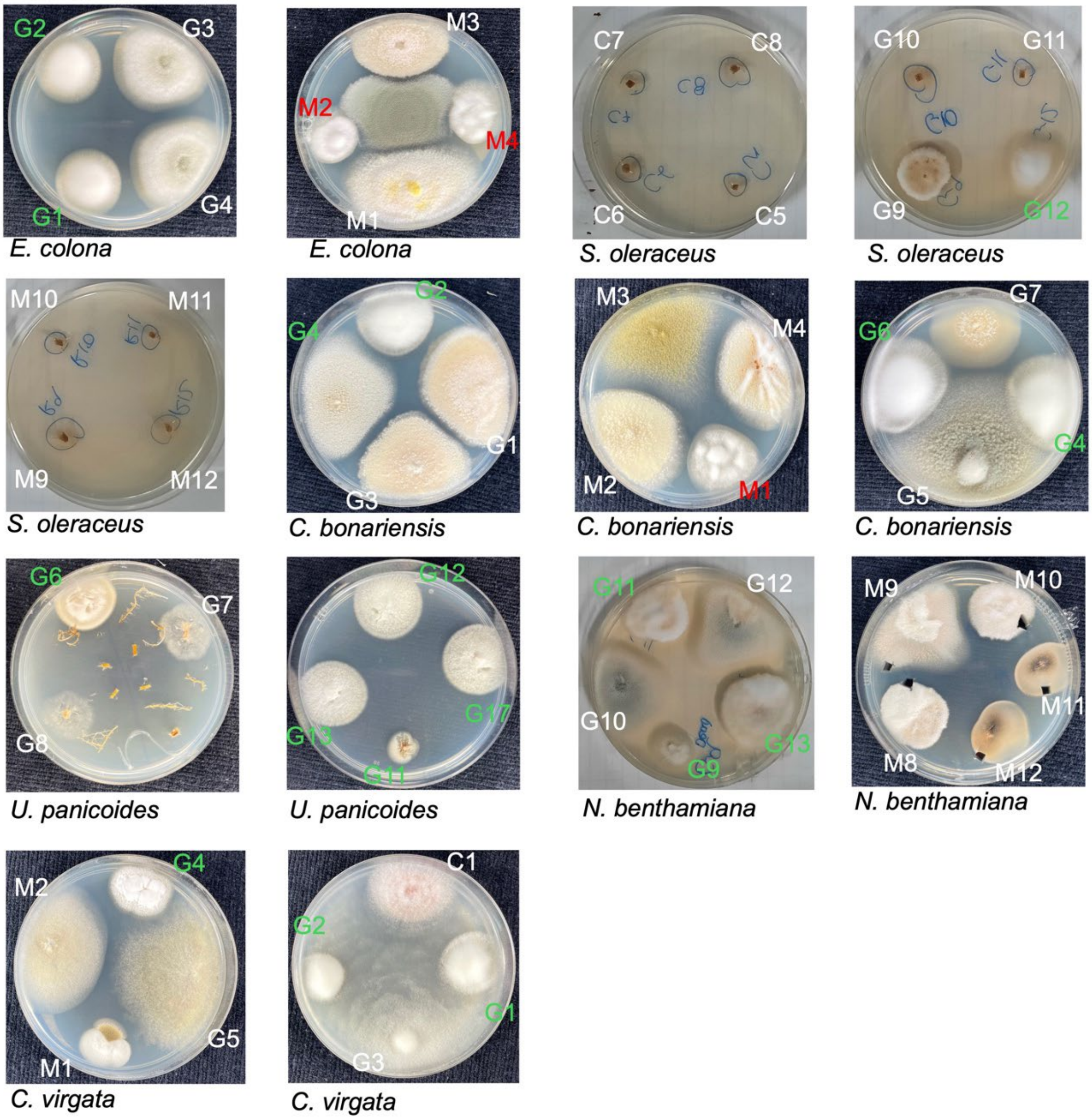

Figure S4. Colonies reisolated from stem sections of weed plant species inoculated with VCG2A-GFP3 or VCG1A-mCherry69. Green = GFP fluorescence confirmed under a confocal microscope. Red = mCherry fluorescence confirmed under a confocal microscope. G = stem issues from plants

inoculated with VCG2A-GFP3. M = stem tissues from plants inoculated with VCG1A-mCherry69. C = stem tissues from uninoculated plants.

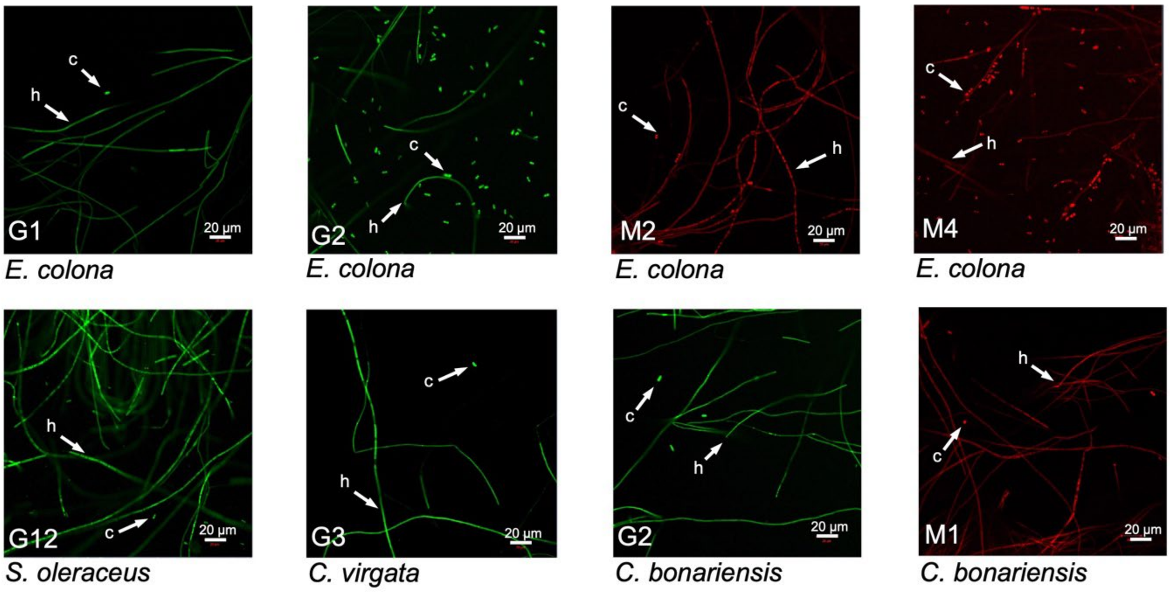

Figure S5. Colonies from stem reisolations confirmed under a confocal microscope to carry GFP or mCherry proteins. Not all positives are presented here. h = hyphae; c = microconidia. bars represent the scale used to capture the confocal images. G = stem issues from plants inoculated with VCG2A-GFP3. M = stem tissues from plants inoculated with VCG1A-mCherry69.

Table S1. Primers for yeast recombinantion-based cloning of to generate a plasmid for expression of mCherry in *Verticillium dahliae*.

Primer Name	Sequence [2]	Target Notes
DG1346 [1]	cctcaccgcggcccatggtctagaactagt**ggatcc**AACGGGC	Forward primer for TEF promoter
DG1347 [1]	gcccttggagaccatGGTGAAGGTTGTGTTATGTTTTGTGGA	Reverse primer for TEF promoter
DG1348 [1]	aacacaaccttcaccATGGTCTCCAAGGGCGAGGAGGA	Forward primer for mCherry
DG1349 [1]	aaatcgaatgtccgcTCATTTGTACAGCTCGTCCATACCG	Reverse primer for mCherry
DG1350 [1]	gagctgtacaaatgaGCGGACATTCGATTTATGCCG	Forward primer for TEF terminator
DG1351 [1]	CTCGAGGTCGACAAGCTTGT	Reverse primer for TEF terminator

[1] Primers used in the cloning of TEF promoter/terminator and mCherry fragments into pPZPnat1. [2] lowercase = homology arms of primer, uppercase = sequence with homology to PCR template, bold = *Bam*HI restriction site.

Table S2. *Verticillium dahliae*-specific primers [31] amplifying a 200 bp ITS product were used to confirm its identity.

Name	Primer Type	Organism	Sequence (5′-3′)	Target
ITS1-F	Forward	Fungi	CTTGGTCATTTAGAGGAAGTAA	18S rDNA
ST-VE1	Reverse	*Verticillium* spp.	AAAGTTTTAATGGTTCGCTAAGA	ITS 1

Table S3. Summary of rate of colonisation based on timing of initial observation at each infection stage throughout the confocal microscopy experiment (At each observation, n = 3 samples of plant tissue section examined).

Infection Stage	First Observed [1] Time		Fungal Structures [2]	Number of Observations [3]
	Sicot	Siokra 1-4		
**Germination**	**24 hpi**	24 hpi	Conidia, germ tubes, infection peg (Siokra)	1 observation
Hyphal elongation	5 dpi	24 hpi	Conidia, hyphae	2 observations
Penetration	5 dpi	24 hpi	Conidia, hyphae	2 observations

Colonisation of the root epidermis	5 dpi	5 dpi	Mycelia	1 observation
Colonisation of the root vasculature	7 dpi	7 dpi	Mycelia, occlusion with conidia (Sicot)	2 observations
Colonisation of the above ground vasculature	-	7 dpi	Conidia, germ tube, hyphae	1 observation
Colonisation of the petiole	-	7 dpi	Conidia, hyphae	1 observation

[1] Time point at which infection stage was first observed. [2] Fungal structures listed were observed on both varieties unless indicated otherwise. [3] Independent observations.

Table S4. mCherry transformant isolates selected for comparison against *Verticillium dahliae* VCG-1A parent, 'Vd71181', that originated from Gwydir Valley, NSW. Isolates were selected for brightness and uniformity of fluorescence. Table includes corresponding Agrobacterium strain used for transformation of mCherry protein into *V. dahliae*.

Isolate[1]	Origin Strain	VCG	Agrobacterium Strain
81T0028	Vd71181	1A	AGL1
81T0029	Vd71181	1A	AGL1
81T0030	Vd71181	1A	AGL1
81T0069	Vd71181	1A	EHA105
81T0073	Vd71181	1A	EHA105

[1] Isolate names are abbreviated to indicate Gw = Gwydir Valley and T = mCherry Transformant.
